# Incorrect Likelihood Methods Were Used to Infer Scaling Laws of Marine Predator Search Behaviour

**DOI:** 10.1371/journal.pone.0045174

**Published:** 2012-10-05

**Authors:** Andrew M. Edwards, Mervyn P. Freeman, Greg A. Breed, Ian D. Jonsen

**Affiliations:** 1 Marine Ecosystems and Aquaculture Division, Pacific Biological Station, Fisheries and Oceans, Canada, Nanaimo, British Columbia, Canada; 2 British Antarctic Survey, Cambridge, United Kingdom; 3 Long Marine Laboratory, University of California Santa Cruz, Santa Cruz, California, United States of America; 4 Department of Biology, Dalhousie University, Halifax, Nova Scotia, Canada; 5 Harvard Forest, Harvard University, Petersham, Massachusetts, United States of America; 6 Population Ecology Division, Bedford Institute of Oceanography, Fisheries and Oceans Canada, Dartmouth, Nova Scotia, Canada; University of Western Ontario, Canada

## Abstract

**Background:**

Ecologists are collecting extensive data concerning movements of animals in marine ecosystems. Such data need to be analysed with valid statistical methods to yield meaningful conclusions.

**Principal Findings:**

We demonstrate methodological issues in two recent studies that reached similar conclusions concerning movements of marine animals (*Nature* 451∶1098; *Science* 332∶1551). The first study analysed vertical movement data to conclude that diverse marine predators (Atlantic cod, basking sharks, bigeye tuna, leatherback turtles and Magellanic penguins) exhibited “Lévy-walk-like behaviour”, close to a hypothesised optimal foraging strategy. By reproducing the original results for the bigeye tuna data, we show that the likelihood of tested models was calculated from residuals of regression fits (an incorrect method), rather than from the likelihood equations of the actual probability distributions being tested. This resulted in erroneous Akaike Information Criteria, and the testing of models that do not correspond to valid probability distributions. We demonstrate how this led to overwhelming support for a model that has no biological justification and that is statistically spurious because its probability density function goes negative. Re-analysis of the bigeye tuna data, using standard likelihood methods, overturns the original result and conclusion for that data set. The second study observed Lévy walk movement patterns by mussels. We demonstrate several issues concerning the likelihood calculations (including the aforementioned residuals issue). Re-analysis of the data rejects the original Lévy walk conclusion.

**Conclusions:**

We consequently question the claimed existence of scaling laws of the search behaviour of marine predators and mussels, since such conclusions were reached using incorrect methods. We discourage the suggested potential use of “Lévy-like walks” when modelling consequences of fishing and climate change, and caution that any resulting advice to managers of marine ecosystems would be problematic. For reproducibility and future work we provide R source code for all calculations.

## Introduction

Technological advances are revealing new insights regarding animal movements in marine ecosystems [1], [2]. Devices attached to animals are becoming smaller in size yet larger in memory capacity [1], and are yielding huge data sets. Given the time, effort and expense devoted to obtaining data from individuals in the marine environment, it is imperative to analyse the data with valid statistical methods. This is particularly important because conclusions concerning animal movement may have management implications [3]. For example, analyses can reveal diel behaviour of critically endangered leatherback turtles during migrations that traverse fishing areas [4], or estimate time spent by Atlantic cod in marine protected areas [5].

One approach to analysing movement data is in the context of Lévy flights and Lévy walks. Lévy flights are random walks for which each movement step is drawn from a probability distribution that has a heavy power-law tail [6]. The original ecological concept [7] was of movement steps being defined as distances between feeding events, although a variety of definitions have since been used [8]. Draws are usually assumed to be independent, such that there is no correlation between consecutive steps and earlier steps do not influence later ones (though see [9]). The power-law tail means that occasionally there will be a very large step. The resulting pattern is of clusters of steps that are connected by the rare long steps. The clusters themselves consist of smaller clusters of even shorter steps connected by longer steps, and so on to give a repeating pattern at multiple scales. Lévy walks are similar, the difference concerns the assumption of time taken to complete each given step, and in ecology these terms have become used somewhat interchangeably [10]. The ecological interest arises from the demonstration that, under certain conditions, a Lévy flight with an exponent of two represents an optimal foraging strategy [11] (and see [12] for further background). Note that such optimality is in the context of random walks with independent and identically distributed step lengths drawn from a power-law distribution, and has recently been shown to be sensitive to assumptions [10].

The first step to identify Lévy movement patterns involves correctly testing whether the movement data are consistent with coming from a distribution with a heavy power-law tail (here, ‘heavy’ means that the distribution has infinite variance). This testing has long been done using regression-based techniques, though these have been shown to be inaccurate and problematic [13]–[16]; for a geological context see [17], [18], and for a general context see [19], [20]. Likelihood methods, a cornerstone of modern statistical ecology [21], have recently been shown to correctly infer exponents of power-law distributions in ecological contexts [15], [16].

Recent work [8] re-analysed 17 data sets from 7 other studies, which had all concluded that the foragers being studied exhibited Lévy flight movement patterns. The foragers ranged in size from microzooplankton [22] to fishermen [23], [24]. The re-analysis, using likelihood methods, overwhelmingly rejected the originally concluded power-law Lévy flight model for 16 out of the 17 data sets when tested against three other simple models. For only one data set (a single grey seal in the North Atlantic Ocean [25]), the data were found to be consistent with coming from a bounded power-law (or truncated Pareto) distribution, which is consistent with a truncated Lévy flight model. However, this does not necessarily then mean that the animal is using a Lévy flight search strategy, and the data set (distances moved in a day) had a sample size of only 71 and only spanned one order of magnitude (7.5 km to 78 km), which limits any interpretation of movement on multiples scales. For further background on the use of Lévy walks/flights in ecology, see a recent book [12] (reviewed in [26]) and review paper [10].

Given the aforementioned results, it is prudent to verify that the techniques applied in related works are valid. Here we investigate the methods used in recent studies concerning movements of marine predators [27] and mussels [28].

In [27], over a million vertical movement displacements were analysed, leading to the conclusion that diverse marine predators (Atlantic cod, basking sharks, bigeye tuna, leatherback turtles and Magellanic penguins) exhibited “Lévy-walk-like behaviour”. This study has been cited 160 times (ISI Web of Knowledge as of 26th April 2012); for further context see [29]. The second study [28] concluded that Lévy walks evolve through interaction between movement and environmental complexity, based on experiments and models concerning movements of mussels (and was followed up by [30]–[32], which we also discuss).

Both studies used likelihood methods to analyse data and reach conclusions. However, we demonstrate three issues with the likelihood calculations; each applies to one or both studies. For clarity, we focus on each study in turn.

Using correct likelihood methods we first re-analyse an example data set from [27] – vertical movements of bigeye tuna. We find no support for a power-law (Pareto) distribution when compared to a simple exponential distribution. This is in contrast to the original finding of close resemblance to an inverse-square power law. This demonstrates that the methodological issues we describe are not just minor technicalities but can yield the opposite biological conclusions to standard methods.

Issue one is that likelihood was calculated in [27] from the residuals of regression fits of models, rather than from the likelihood equation of the underlying probability distribution being tested. Such regression fits result in the testing of models that do not correspond to normalised probability distributions (Issue two). This approach resulted, for [27], in the conclusion of overwhelming support for a “quadratic” model (for the bigeye tuna data and for four of the other six species). Yet we show that the quadratic model is spurious because its probability density function has negative values (Issue three); it also has no biological justification.

The results of our re-analysis of the bigeye tuna data contradict the original conclusions for those data. The problems identified here with the original methods of [27] consequently question the original results for the other data sets and thus question the central conclusion of “scaling laws of marine predator search behaviour”. Note that we have not re-analysed the remaining data sets in [27], and so do not make definitive conclusions regarding them.

We then describe some methodological issues of [28] and demonstrate how likelihood was also incorrectly calculated from regression fits (Issue one). Re-analysis of the data finds that although a truncated Lévy walk is more supported by the data than an alternative exponential model, it is decisively rejected by goodness-of-fit tests as being a suitable model. Thus we do not agree with the original conclusion of Lévy walk movements of the mussels.

We also discuss some aspects of the methods in another study [33] that analysed marine predator movements. We end by showing that Issue one also occurred in a recent example from terrestrial ecology [34], which concluded an exponential model was preferred over a Lévy model. Thus, the issues we present are not restricted to studies of marine animals, or to those that support the Lévy idea.

The issues we demonstrate reinforce that likelihood, as with all methods in ecology, must be used properly, and that claims of Lévy movements by animals do not always hold up to scrutiny. The prevalence of important methodological errors in high-profile papers that test for Lévy movement patterns is problematic, leading to incorrect biological conclusions. This negatively impacts the general field of movement ecology, and could have undesirable consequences if conclusions from such studies influence management decisions concerning marine ecosystems.

All computations used R version 2.9.2 or later [35]. To allow other researchers to more easily use our methods in the future and reproduce all our results, we provide R source code (see Supporting Information) for all calculations and figures, a practice recommended by [36].

## Analyses and Results

### Marine Predator Movements in Ref. [27]

In [27], electronic tags were attached to marine predators, resulting in over a million vertical movement displacements. The principal result was that, for five species, model fits of the frequency distributions of vertical movements “closely resembled an inverse-square power law with a heavy tail of increasingly longer steps intermittently distributed within the time series that is typical of ideal Lévy walks” [27]. The five species were Atlantic cod, basking sharks, bigeye tuna, leatherback turtles and Magellanic penguins. The inverse-square power law relates to the aforementioned theoretical optimal foraging strategy, and it is striking that movements of such diverse predators should closely follow such a power law. For the two other species tested, catsharks and elephant seals, “Lévy-like” processes were not concluded. However, here we demonstrate three problems with the likelihood methods used to obtain the results, and thus question the overall conclusions.

We first re-analyse the bigeye tuna (*Thunnus obesus*) data set from ref. [27]; data (all three individuals pooled together) courtesy of D. Sims. The data set consists of 29,900 vertical displacement steps, defined as follows: “the change in selected water-column depth between consecutive time intervals, 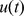, was calculated to derive a time series of vertical displacement (move) steps for each individual” (Methods section of [27]). Steps were measured in metres, with each time interval being 1 minute. The bigeye tuna data set appears to be the largest of the data sets analysed in [27] (vertical axes of Supplementary Figure 1 and page 4 of Supplementary Information of [27]).

**Figure 1:**
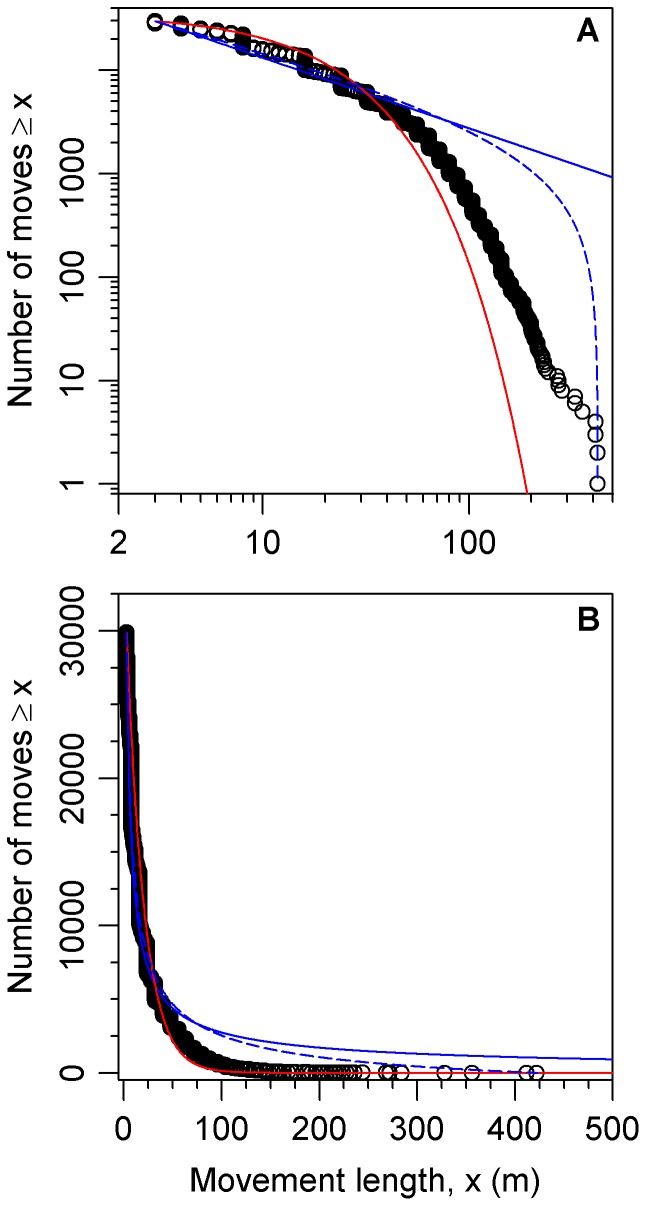
Rank/frequency plots of bigeye tuna data with distributions fitted here using likelihood. (A) Logarithmic axes. Black circles are the 29,900 data points, as shown in Supplementary Figure 1(h) of [27]. The four distributions fitted here are power law (blue straight line), exponential (red curved line), bounded power law (blue dashed curved line), and bounded exponential (red dashed curved line, indistinguishable from exponential). (B) As for (A), but on linear axes to eliminate distortion due to the logarithmic axes. Our Akaike weight analysis found the exponential distribution to be the most supported model, but goodness-of-fit tests, using the two alternative binning methods described in [8], both yield 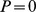 (with respective degrees of freedom of 82 and 6 and goodness-of-fit values of 41,532 and 4,589). Thus the data are not consistent with the exponential distribution.

To compare models in [27], “The relative likelihoods of candidate models were calculated using 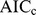 weights [37]” (page 1 of Supplementary Methods of [27]), where 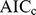 is the small sample Akaike Information Criterion. This would indeed appear to be the logical way to compare candidate statistical models [14], [37].

However, the resulting 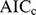 weights (henceforth termed Akaike weights) were calculated using four methods (described below), yielding four sets of results. Akaike weights are based on likelihood functions, and the models being tested are simple probability distributions with one unknown parameter. Therefore the likelihood functions are uniquely defined and have analytical solutions for maximum likelihood estimates, and so we did not understand the need for multiple methods.

Note that two further methods (involving root-mean square fluctuations and power-spectrum analysis) were used in [27] to test for the presence of long-term correlations that may also characterize scale-invariant Levy walks, but here we focus on the methods that were used to fit power-law distributions of movements, determine the power-law exponent 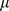, and compare with alternative distributions.

#### Re-analysis using standard likelihood methods and Akaike weights

First, we compare the support for four models using likelihood functions. Full R code for these calculations is given in the Supporting Information (code S1 and code S3). The models and corresponding probability density functions 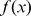 for movements of length 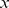, are [8], [14]: (i) the classic Lévy flight model of an unbounded power-law tail (PL model)
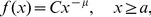
(1)with exponent 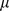, minimum movement length 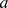 and normalisation constant 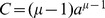; (ii) the simplest alternative of an unbounded exponential tail (Exp)
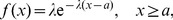
(2)with parameter 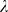; (iii) a bounded power law (PLB)
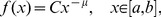
(3)where 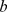 is the maximum allowable value of the data for the bounded models and normalisation constant 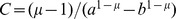 for 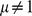 and 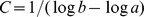 for 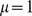 (see [8]); (iv) a bounded exponential distribution (ExpB)
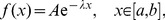
(4)with normalisation constant 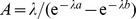.

The Lévy flight hypothesis is that the distribution of movements has a power-law tail with 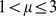. This is the PL model (1), and the hypothesis is not directly concerned with data that are 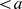. The PL model with 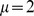 corresponds to the inverse-square power-law that [27] found close resemblance to for five species. The exponential distribution (2) represents the simple hypothesis that each movement step terminates with a constant probability per unit time [16], [38]. Ref. [27] found an exponential distribution to be supported for only two species (catshark and elephant seal).

The bounded versions of the two distributions are tested here due to previous lack of support for the unbounded power-law model [8], [14], [16]. For the two bounded models, the upper bound *b* was set to the maximum movement length. For all models, the lower bound *a* was set to the minimum movement length (as assumed by [27]). Note that for the PLB model (3), 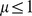 is permitted (unlike for the PL model (1)), and that 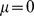 gives the uniform distribution.

We use the unique likelihood functions of the respective probability distributions to find the maximum likelihood estimates for the parameters, which are used to plot the distributions and compute standard Akaike weights [37]. The log-likelihood functions are explicitly derived as equations (5) and (6) in [14], and equations (A.23) and (A.27) in [8]. The equations are based on standard likelihood theory [37], [39]. The Akaike weight calculations are also given in [14]. The Akaike weight for a model is considered as the weight of evidence in favour of that model being the best model for the given data set, out of the models considered. By definition, Akaike weights for the tested models sum to 1. We also perform a goodness-of-fit test on the best model to see if it is indeed a suitable descriptor of the data [14], [40], [41], using the methods described in [8].

Figure 1(A) shows the bigeye tuna data set plotted as a rank/frequency plot on logarithmic axes; Figure 1(B) is the same plot on linear axes. Supplementary Figure 1(h) of [27] is such a plot also on logarithmic axes (though the model fits, discussed shortly, are different). Such logarithmic axes are used in power-law studies because data from a power-law distribution would appear straight (with some curvature in the tail, e.g. Figure 1(d) of [16]).

The distributions shown in Figure 1 use the respective maximum likelihood estimates for the parameters. None of the models appear to fit the data particularly well, especially for movements 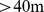. The power-law models over-estimate the magnitude of longer moves (the blue curves decay away too slowly), whereas the exponential models under-estimate them (the red curves decay away too fast); though bear in mind that the bulk of the data set comprises movements 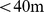.

From the maximum likelihood estimates, we calculate standard Akaike weights [14], [37]. We find the power-law distribution has no support (Akaike weight of 0) compared to the exponential distribution (Table 1, method **a**). For ease of comparison with the results of [27], that did not consider bounded models, in Table 1 we only present our calculated Akaike weights for the unbounded models; when comparing all four models in the order given in (1)-(4), the Akaike weights are 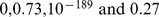, such that bounded power law also has no support (Akaike weight of 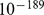).

**Table:** **Table 1.** Akaike weights for North Pacific bigeye tuna data.

Method	Power-law model	Exponential model	Quadratic model
**a**, Maximum likelihood (calculated here)	0	1	–
**b**, Supplementary Table 3 of [27]	0.769	0.231	–
**c**, Supplementary Table 4 of [27]	>0.999	<0.001	–
**d**, Supplementary Table 5 of [27]	>0.999	<0.001	–
**e**, Supplementary Table 6 of [27]	<0.0001	<0.0001	∼1.000
**f**, As for **e** but no quadratic model	0	1	–

**a**, Properly defined Akaike weights [37], calculated here from the raw data (all individuals pooled together) using the equations in Box 1 of [14]. Respective log-likelihoods are 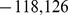 and 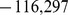, giving Akaike Information Criteria of 236,256 and 232,599. **b**, Data for each individual were binned using the log-binning with normalization (LBN, [13]) technique, and regression lines fitted to all the points plotted on one figure (see Supplementary Fig. 3 of [27]). **c**, LBN method for all individuals pooled together [27]. **d**, LBN method with generalised linear mixed-effect models, using individual as a random factor [27]. **e**, Bayesian (rather than Akaike) Information Criteria [37] weights based on fitting linear regressions to rank/frequency plots [27] for all individuals pooled together. **f**, Same method as **e** but calculated here for just two models (result can also be deduced from Supplementary Table 7 of [27]).

Our result contradicts the Akaike weights calculated in [27], which were derived using four methods (denoted **b** - **e** in Table 1). Methods **b** - **d** in Table 1 involved fitting regressions to logarithmically-plotted binned data, with **c** and **d** concluding overwhelming support for the power-law model over the exponential (Table 1). A “quadratic model” was introduced for the rank/frequency method (**e**). All methods tested the models over the full range of the data (e.g. Fig. 1 of [27]), as we have done here. The contradictory weights arise from three issues that we illustrate below for the rank/frequency method (**e**).

Note that Methods **b** and **d** involved considering the three individual tuna separately – given that we used the pooled data our results are directly comparable to those for methods **c**, **e** and **f** (though our methods could be applied to the individual data sets). However, the issues that we identify hold for all methods. Also, for method **e**, Bayesian, rather than Akaike, weights were calculated in [27], but this is tangential to the issues we now describe (see *Methods*).

#### Issue one: likelihoods were computed from linear fits of models, rather than from the underlying probability distributions being tested

For the rank/frequency method (Table 1, method **e**) movement steps, *x*, were put in descending order such that their respective ranks were given by 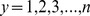; 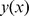 thus represents the number of steps 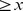. The exponential model was tested by fitting a straight line to 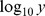 against *x* (page 4 of Supplementary Information of [27]). Thus,
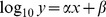
(5)where 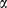 and 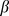 are the fitted coefficients. For the tuna data (sample size 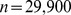), we obtain 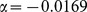 and 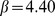 using linear regression, and compute a log-likelihood of 29,016.7 using the 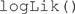 function in R [35]. This reproduces the log-likelihood value in Supplementary Table 7 of [27]. Whether this is the exact approach used in [27] could not be confirmed by the authors when queried, but our calculation exactly agrees with the reported value. We also exactly reproduce the other two log-likelihood values reported for bigeye tuna in Supplementary Table 7 of [27]. Full R code for Issues one to three is given in the Supporting Information (code S2).

However, this log-likelihood calculation is based on the standard assumption of Gaussian errors when fitting a straight line. Since 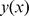 are ranks 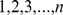, the interpretation of such errors is problematic. More importantly, the resulting log-likelihood corresponds to the likelihood of the observed residuals around the fitted straight line assuming a Gaussian residual model, rather than the likelihood of the observed data coming from the exponential probability distribution (which is the hypothesis being tested). The resulting log-likelihood depends on the sum of squared residuals around the fitted line, given on page 12 of [37] as
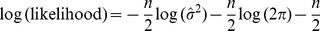
(6)where 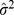 is the maximum likelihood estimate of the variance of the assumed Gaussian errors and is given by 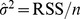, where RSS is the residual sum of squares of the errors (see also page 172 of [39]).

Calculations from the data give 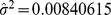, yielding a log-likelihood from (6) of 29,016.7, matching the value given by [27] and the aforementioned value calculated using the R function 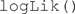. Inspection of the source code of 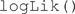, by typing 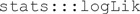 in R, confirms that it does use (6) to give a log-likelihood value. This is the correct approach if testing a *functional relationship*, whereby 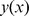 is a function of *x* (and Gaussian errors are assumed). But the situation here requires the testing of a *probability distribution*, whereby 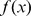 is the probability density function of *x*.

Thus, 29,016.7 is not the log-likelihood of the exponential *distribution*, which we calculate to be 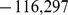. The latter is what should be used when computing Akaike (and Bayesian) weights to compare probability distributions [37]. Using this value, with the corresponding value of 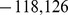 for the power-law distribution, gives the aforementioned Akaike weight of 1 for the exponential model, and no support for the power-law model (Table 1).

#### Issue two: the tested models are not normalised probability distributions

The above regression approach is fitting a model for which the associated probability density function is
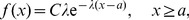
(7)where *a* is the minimum value of x, 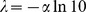 and 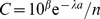. We know that 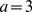 from the data. The derivation of (7) is given later in *Methods*.

Equation (7) requires 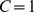 to be a correctly normalised exponential distribution; otherwise 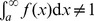. However, the regression calculation gives 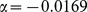 and 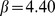, leading to 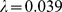 and 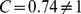. There is no constraint on the regression coefficients 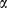 and 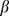 to correctly normalise the probability density function such that it integrates to one.

Graphically, this can be seen in Fig. 2c of ref. [27] and Supplementary Fig. 1 of ref. [27] – the red curves representing the fitted exponential distributions do not start at the left-most data point. For correctly normalised distributions they would, because the number of predicted values 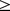 the minimum data value will, by definition, equal the sample size; this can be seen for all the estimated distributions in our Figure 1. For the aforementioned Fig. 2c of [27], the fitted distribution predicts only ∼400 values 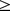 the minimum value, but the data set consists of 1025 such values.

**Figure 2:**
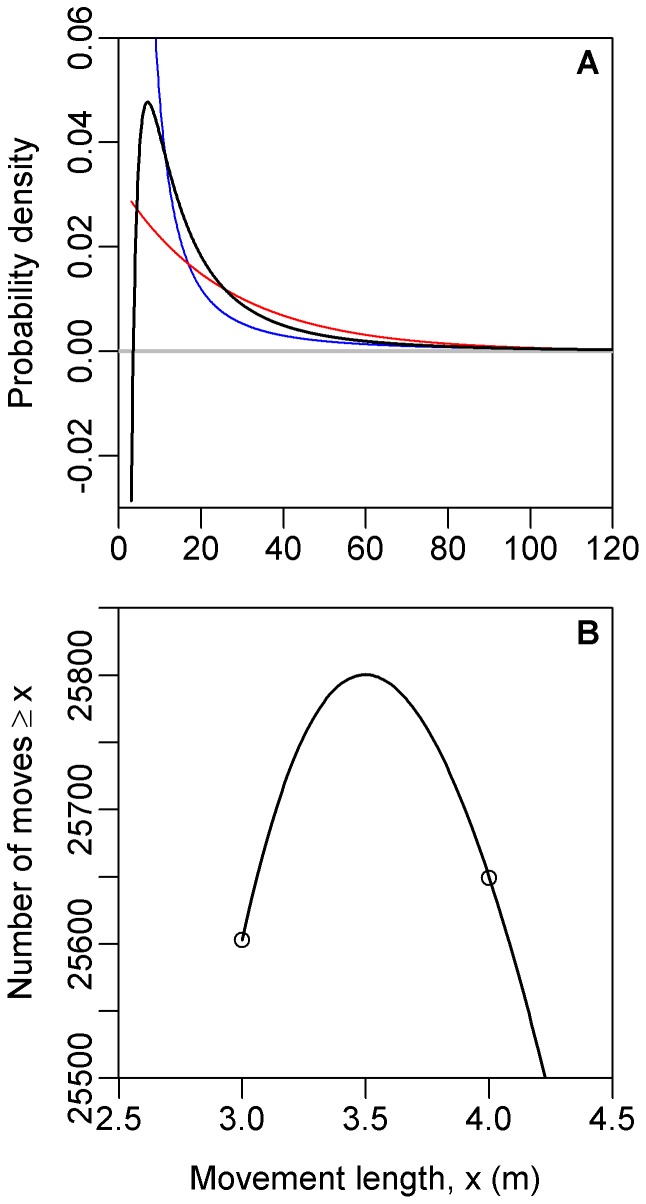
Probability density functions for the bigeye tuna data, corresponding to the model fits calculated using regression in [**27**]. (A) Functions start from the value 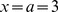, the minimum value of the data. Blue is the power-law model (it reaches 0.52 at 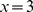), red is the exponential and black is the quadratic model given by (8). The density function for the quadratic model goes negative, violating a fundamental requirement of probability density functions. (B) Plotting the quadratic model on the same axes (though magnified) as Figure 1(B) further demonstrates the issue. For example, as highlighted by the circles, the model erroneously predicts more movements 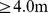 than 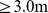, a clear contradiction.

So the reported log-likelihood from the incorrect regression method (reproduced above) relates to a function that is not an exponential distribution. The estimated value of 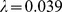 differs from the correct maximum likelihood estimate (e.g. [14]), which is simply 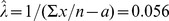.

#### Issue three: the quadratic model obscured support for the exponential model over the power-law model

The weights for the power-law and exponential models were not directly compared for the rank/frequency method [27], yet they were for the other methods. Instead, on page 4 of the Supplementary Information of [27], “a quadratic model 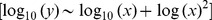 describing intermediate behaviour” between the power law and exponential was introduced. All three models were compared, rather than just the exponential and power law. The quadratic model was found to be overwhelmingly supported for the tuna data (using Bayesian weights; Table 1, **e**) and for five of the other seven movement data sets (Supplementary Table 6 of [27]), yet was not referred to in the main text of [27].

However, this model also corresponds to an invalid probability density function. Similar calculations to those described for (7) give the resulting probability density function
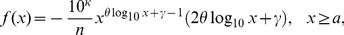
(8)obtained by writing 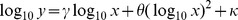 to fit the quadratic model on 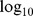 axes, where 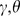 and 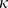 are the regression coefficients; see the *Methods* for the full derivation. Multiple linear regression [21] gives 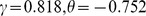 and 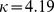, (and the residual-based log-likelihood of 40,526.4, reproducing that in Supplementary Table 7 of [27]). These coefficients give 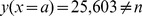, showing that (8) is not a normalised probability density function.

The curves given by (7) and (8) (and (9) which is defined below) are plotted in Figure 2, to see how the models compare when plotted as probability density functions. Given the quadratic model’s highly nonlinear formulation (8), we anticipated that it might have a hump shape, unlike the decreasing power-law and exponential functions. However, Figure 2 shows that it actually takes negative values. The negative values violate the fundamental property that a probability density function must be 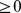. An analogy would be to say that a tossed coin has a probability of 0.5 of landing heads, and a probability of 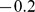 of landing tails.

Figure 2(B) magnifies the start of the quadratic function plotted on rank/frequency axes (blowing up the start of Supplementary Figure 1(h) of [27], but without logarithmic axes). The negative density function means that the model predicts 25,603 moves 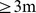, yet almost 200 more (25,801) moves 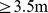, and then 25,649 moves 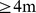. Obviously, a model should not predict more moves 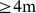 than it does moves 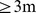. The fundamental reason that this problematic situation arose is that a quadratic function, which is hump-shaped, was fitted to a rank/frequency plot, which by definition cannot be hump-shaped (it must be non-increasing).

We agree that the quadratic model “has no particular statistical or biological justification” (page 4 of Supplementary Information of [27]). Without it, we find that the erroneous rank/frequency method of [27] actually favours the exponential model (Table 1, **f**) for the tuna data. This conclusion was obscured by the introduction of the third (quadratic) model.

Had the quadratic model been a valid model (i.e., a properly normalised non-negative probability density function), and been justified as an intermediate model between the power law and exponential models, then the support found for this model should also have implied no support for the Lévy hypothesis (because it is an intermediate model). However, [27] found the quadratic model to be the best supported for all data except for two of the predators, and said that this indicated “intermediate behaviour and Lévy-like movement as assessed using rank-frequency plots” (page 5 of the Supplementary Information of [27]).

Issues one and two also apply to the power-law model. Setting 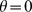 in (8) and substituting 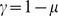 (where 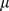 is the traditional power-law exponent) gives
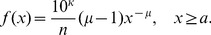
(9)


To be the normalised power-law probability density function (1) requires 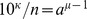, and hence there should only be one estimated parameter, 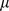. Again, there is no reason why the regression coefficients 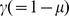 and 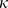 should give the correct normalisation. This seems to be an additional, yet generally overlooked, problem with using such regression methods to estimate power-law exponents – see [10] for a slightly different way of thinking about such issues.

By attempting to reproduce the original results we realised the regression intercept parameter, 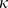, was used in such calculations – we have not seen it explicitly used in other power-law related studies (see [10] for the implicit consequences). The simple solution to this and the other regression-based issues is to use the unique maximum likelihood estimate of the power-law probability density function [14]–[16], [20], as done here.

Issues one and two also apply to the binning methods (**b-d**) of [27] – likelihoods were incorrectly calculated and tested models are not normalised probability distributions. These issues are in addition to the inaccuracies known to occur when using such regression approaches to estimate power-law exponents [13]–[16], [20]; also, goodness-of-fit was not properly assessed [14], [16], [40], [41]. Thus, distributions were tested erroneously throughout [27], and the original result of close resemblance to “an inverse-square power law … that is typical of ideal Lévy walks” [27] was based on incorrect methods.

### Mussel Movements in Ref. [28]

To conclude that Lévy walks evolve through interaction between movement and environmental complexity [28] first requires demonstrating that the animals in question are using a Lévy walk to move. In [28], step lengths of mussels were “estimated by the distance between two subsequent reorientation events”. Movements were analysed as follows: “the fit to the step length data of solitary mussels was calculated using Maximum Likelihood estimation by fitting the inverse cumulative frequency distribution to that of the experimental data.” (line 92 of the Supporting Online Material of [28]). (Such an ‘inverse cumulative frequency distribution’ is also known as the survival function, and is essentially what we show in Figure 1 but with the y-axis scaled by sample size so that it goes up to 1).

The unnecessary specification of a plotting method when using likelihood suggests that some of the aforementioned problems may again be applicable. Ref. [28] continues “By comparing the inverse cumulative distributions to that of the data, Goodness-of-fit (*G*) and the Akaike Information Criterion (AIC) were calculated as well as the variance explained by the fitted model (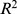).” This further suggests that likelihood (and therefore AIC) was incorrectly calculated, and that 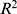 calculations continue to be inappropriately used in Lévy studies (see [16]), prompting us to investigate the details of the methods used.

Examination of the detailed Supporting Online Material of [28], email clarifications with the lead author, and examination of the R code used for the analyses (M. de Jager, pers. comm.), determined the methods used to estimate parameters, calculate likelihoods and compare the fits of models. These are documented below in *Methods*, together with identification of several problems, the most relevant of which we now summarise.

For the bounded power-law (PLB) model (3), only discrete values of the exponent 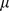 were tested. This limits the accuracy of the method, and does not allow for calculation of confidence intervals to characterise uncertainty. Also, multiple values of the upper bound 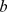 were tested to maximise the likelihood. However, this is not needed, because simply setting 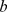 to be the maximum value in the data set will maximise the likelihood.

Issue one occurs – AIC calculations were again based on linear fits of models. This is because AIC was calculated in R using the command
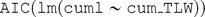
where 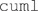 is the observed distribution and 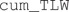 is the fitted distribution. The 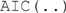 command calculates likelihood from the linear regression 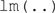, rather than from the underlying probability distribution being tested.

A Rayleigh distribution was also analysed in the R code from [28], and “used for Brownian motion”, although this is not mentioned in [28], which specifically says in the opening paragraph that step lengths “are derived from an exponential distribution in the case of Brownian motion”. This latter quote relates to a misunderstanding that we return to in the *Discussion*.

#### Re-analysis of mussel data

In a recent Erratum [30], published on 23rd December 2011, the authors have somewhat addressed the above concerns. These concerns, and several others (most notably that the data set was problematic), were brought to their attention by V. Jansen and F. Van Langevelde (independently of us). The authors acknowledged that Issue one occurred, and presented a replacement of their original Fig. 1B with model fits calculated using the likelihood methods of [14]. We have independently reproduced this figure and the model fits (using the corrected data set), and confirmed agreement (M. de Jager, pers. comm.) of our estimates for the power-law exponents 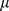 for the Lévy walk and truncated Lévy walk models (PL and PLB models in our terminology). Full R code for this section is given in the Supporting Information (code S3 and code S4).

We agree that the truncated Lévy walk model is indeed more supported by the data than an exponential model (overwhelmingly so, given our calculated respective Akaike weights of 1 and 0). However, as emphasised by [40], we also performed goodness-of-fit tests [42] in [14], to test if the data are consistent with coming from the favoured model. While one model may indeed be favoured over another, it still might not be a suitable model – see also [8], [41].

We therefore conduct goodness-of-fit tests on the corrected mussel data set. Our results decisively reject (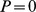) the hypothesis that the data are consistent with coming from the truncated Lévy walk (PLB) model shown in the Erratum [30] (see *Methods* for details). Thus, we do not agree with the Erratum’s finding that the “overall conclusion of the [original] paper that mussels adopt a Lévy walk … remains unchanged”.

In February 2012, a Technical Comment [31] on [28] was published, with a Response by the original authors [32]. Ref. [31] noted that theory, knowledge that mussels can switch between moving very little (or not at all) and moving much farther, and visual inspection of the data, suggested testing of a composite Brownian walk (whereby mussels switch between different modes, in each of which they display Brownian motion). To test this, [31] used sums of two, three or four weighted exponential distributions, and used AIC to compare support for these models with the original three models used in [28] (Exp, PL and PLB). The resulting Akaike weights most supported the three-exponential model of composite Brownian motion. They found that the truncated power-law (PLB) model is supported over the exponential only if the composite Brownian models were not included.

In their Response [32] to [31], the original authors re-analysed their (corrected) data by fitting models to movements of the eight individual mussels that had a sample size 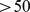 (rather than grouping all data together into one data set, as done originally). Five models were tested, the original three plus composite Brownian walks consisting of sums of two or three weighted exponentials (following [31]). Referring to their Table 1 and Figure 2, they stated that “A truncated Lévy walk provided large improvement over a Brownian walk,” – their Table 1 shows that for six of the eight mussels the AIC for the truncated Lévy walk is lower than that for the Brownian walk. The authors continue “whereas a composite Brownian walk provided only small further improvement in fit,”.

However, their Table 1 does not support this statement – the composite Brownian walk models give much better fits than the truncated Lévy walk model. (The one exception is mussel B, for which the simple Brownian walk gives the best fit anyway). The Akaike weights for the truncated Lévy walk model are 0.000 for five mussels, and 0.002, 0.003 and 0.054 for the remaining three. The Akaike weight of 0.054 corresponds to mussel F – the evidence is thus “reasonably strong” [37] against the truncated Lévy walk being the most suitable model. Yet for the remaining seven mussels the Akaike weights for the truncated Lévy walk model are so small that we conclude that the simple or composite Brownian walks are overwhelmingly supported compared to the truncated Lévy walk model, in contrast to providing the reported “only small further improvement” [32].

### Marine Predator Movements in Ref. [33]

In [33], strong support was found for “Lévy search patterns across 14 species of open-ocean predatory fish (sharks, tuna, billfish and ocean sunfish), with some individuals switching between Lévy and Brownian movement as they traversed different habitat types.”. Vertical dive data were again analysed to reach conclusions of one-dimensional Lévy or Brownian walks, after first dividing long time series of vertical movements into shorter sections using a split moving-window analysis. A total of 129 sections were analysed, of which 35 were determined visually to be poorly fitted by the candidate distributions, leaving 94 sections to be analysed statistically. Also, georeferenced locations that indicated animals’ locations were overlaid on, for example, satellite maps of chlorophyll *a* concentrations, which we agree is a valuable endeavour. The only data that had been originally analysed in [27] were for basking sharks. Note that new bigeye tuna data were analysed in [33], and it was concluded that for 19 out of the 32 sections, a truncated power-law provided the best fit.

Ref. [33] did not use the aforementioned methods of [27]. Their methods were based on those developed and tested more recently in [43]. Ref. [43] developed a method to estimate, for the PL model (1), the most suitable value of *a* to be considered as the start of the tail. However, [33] used this approach to also estimate *b* (their 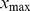) for the PLB model (3), the maximum value of the data to be fitted to by the model. This was often less than the maximum value of the data set. To see this, compare the ‘Max step length (m)’ column with the ‘Best fit Xmax’ column in Table S3 of [33]. The first example is for bigeye tuna 1 (section 2), for which the maximum step length in the data was 1,531 m but the best fit 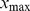 was only 466 m. Thus, for this example (which happens to be the most extreme), step lengths 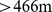 were not part of the final model fits, even though values up to 1,531 m were recorded. Of the 94 data sets (sections) analysed statistically, 66 were best fitted by the PLB model (compared to other models). Of these 66, 28 (42%) have ‘Best fit Xmax’ less than the ‘Max step length’ of the data. The 28 cases have a mean ratio of ‘Best fit Xmax’ to ‘Max step length’ of 0.75, with five-number summary (minimum, quartiles and maximum) of 0.30, 0.59, 0.80, 0.91, 0.99 (Supporting Information Code S5).

So [33] tested bounded power-law distributions, which we have also done (e.g. here and in [14]). However, when doing so we fixed the upper bound *b* to be the maximum data value (or higher [14]), because the Lévy flight hypothesis is concerned with the rare longer steps in the heavy tail of the data. As [33] say when introducing their work, “Lévy flights describe a movement pattern characterized by many small steps connected by longer relocations”, with the probability density function of steps having “a power-law tail in the long-distance regime”. However, the lower and upper bounds were fitted to “find the distribution that best fit most of the data” (page 13 of Supplementary Information of [33]), rather than biological reasons such as, for example, if the largest movements are known to be diving associated with thermoregulation. In our opinion, to fit a model that results in often ignoring the longer steps in the tail of the data seems somewhat at odds with the very Lévy flight hypothesis being tested. Furthermore, it is known that some probability distributions (such as the lognormal) can, when looking at restricted ranges, be mistaken for power laws [44].

Related to this, [45] gave a rule of thumb that “a candidate power law should exhibit an approximately linear relationship on a log-log plot over at least two orders of magnitude of data in both the *x* and *y* axes”. Indeed, the original Lévy flight hypothesis [7] of a pure power-law distribution was deduced to hold over almost two orders of magnitude, based on such a linear relationship. But of the 66 aforementioned sections that were best fitted by the PLB model in [33], only 7 occurred over two orders of magnitude (i.e. in Supplementary Table S3, for only 7 cases is the ‘Best fit Xmax’ 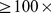
‘Best fit Xmin’ when the PLB model is the best fitting distribution). So of 129 data sections originally analysed, only 7 found a bounded power-law over at least two orders of magnitude to be the best fitting distribution. Thus, a bounded power-law distribution may indeed be the most suitable model for a data section, but if this range is less than two orders of magnitude (as is usually the case in [33]), we question how strongly this represents evidence for the Lévy flight hypothesis, part of whose appeal involves movement patterns being invariant across multiple scales.

### A Terrestrial Example

The issues we have highlighted are not solely confined to work whose conclusions support the Lévy idea, or to marine ecology. Recently, [34] analysed movements of Australian desert ants, concluding that the data did not show characteristics of a Lévy walk strategy. The methods of [14] were used to compare the PL and Exp models across the tails of the data, concluding that the Exp model was preferred (Table 2 of [34]). However, the data were considered to be rather poorly described by the Exp model, but much better described by fitting two separate functions to the short and long ranges of the distribution (Table 1 of [34]). The resulting fits were compared using AIC “based on the residual error” of regression fits [34]. This is again related to Issue one described above. The solution here would be to explicitly write down the probability density function being tested and then work out the likelihood function (as since done in [31]).

## Discussion

We have identified three methodological issues that each occurred in one or more recent studies. The studies made similar conclusions regarding animal movements. Likelihood was calculated incorrectly in [27], [28], leading to incorrect AIC calculations, and thus to invalid conclusions regarding model selection, and consequently to misleading biological conclusions. In particular, we have shown that one issue, of likelihoods being computed from linear fits of models rather than from the underlying probability distributions being tested, has occurred in slightly different ways in three papers [27], [28], [34]. This method is not merely inaccurate, it is fundamentally incorrect.

When applying proper likelihood methods to an example data set from [27], the original results for the data set are overturned. This demonstrates that the methodological issues are important, questioning the original central conclusion of “scaling laws of marine predator search behaviour” that was based on the incorrect methods. Since we have not re-analysed all the data sets from [27], we do not claim to have overturned all the original conclusions (concerning all the data), rather we question them because they were based on methods shown to be incorrect. A full re-analysis using correct methods may indeed reach the original conclusion for some of the data sets of close resemblance to an inverse-square power law over the full ranges of data. Likelihood problems were demonstrated with the methods of [28], and re-analysis of the data rejects the study’s central conclusion that mussels use a Lévy walk movement strategy.

Although we found the power-law distribution to have no support compared to the exponential for the bigeye tuna data set of [27], we do not claim that the exponential is a suitable model (as seen in Figure 1 and the associated goodness-of-fit results). Rather, more complex behavioural models [3], [46] are likely required to understand these data (as was indeed acknowledged in [27]). Whether such models could be described as “Lévy-like” [27] would be hard to evaluate, because this term was never defined. This restricts quantitative inference of how “non-Lévy-like” a pattern has to be to not be considered “Lévy-like” (see also [29]). Also note that the results have since been interpreted as standard Lévy flights [47] rather than the somewhat weaker “Lévy-like”.

With regard to the exponential distribution, there seems to be a misunderstanding concerning Brownian motion. We previously [8], [14], [16] tested the power-law distribution against the exponential distribution because the exponential represents the simplest alternative hypothesis of steps arising from an uncorrelated Poisson random process. The exponential distribution was one of the alternative distributions considered in [48] because “It can be shown that if the probability per unit length to terminate the walk remains constant”, i.e. a Poisson process [38], then “the distribution of lengths of many walks has an exponential form.”. As [22] stated: “Exponential laws, through the Central Limit Theorem, give rise to asymptotically Gaussian statistics (Brownian motion)”. And [49], referring to simulating a random walk using the PL model (1), stated “If 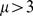 the movement process is a Brownian random walk.”.

The above examples are correct and consistent with each other. The Exp model (2) represents a simple hypothesis. It gives rise to Brownian motion, as does the PL model (1) with 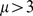 (because the distributions have finite variance). However, since any distribution with finite variance would also give rise to Brownian motion in the long-term limit [50], to rule out Brownian motion it is not sufficient to just rule out the Exp model – the Exp model is just the simplest model. However, see below for other modelling approaches.

We explained a concern regarding the estimation of lower and upper bounds of the tested distributions. This raises a fundamental issue that the whole idea of Lévy flights is only concerned with the *tail* of the data. A data set may indeed need some pre-processing before being analysed (say, to exclude measurements that are not representative of the biological process being studied). But any model should really be fit to the complete resulting data set. To test for a power-law tail it would be better to fit, to the resulting data set, a distribution that spans all the data and has a power-law tail, rather than to use a method that decides where the tail starts for each model and ignores smaller data values.

As noted in the *Introduction*, correctly testing whether the movement data are consistent with coming from a distribution with a heavy power-law tail is only the *first* step in identifying Lévy movement patterns. If this first step results in a positive result of a heavy power-law tail, it is not appropriate to then directly conclude that the animals are actually using such a movement strategy to search for food, as discussed in a recent review [10]. For example, the observed data may not directly correspond to actual complete straight-line animal movements between changes of direction, as usually assumed in Lévy analyses, and such sampling issues can affect results [40], [41], [51]. Also, Lévy and Brownian motion models are simple descriptions of animal movements, whereas the actual strategies used by animals will involve memory, intelligence and intermittent strategies [10]. And animals move for reasons other than foraging for food, yet simple descriptive statistical analyses ignore any role of behaviour.

One solution to the aforementioned problems is in the framework of mechanistic state-space models [3], [52]. This requires coupling of an explicit observation model with a biological movement model, separating the procedure of observing the animal (that yields the data) from the movements that the animal is actually making. Movement can then be partitioned into different behavioural states, such as ‘searching’ and ‘migrating’. The time series in [33] were long enough to enable separation into shorter sections, which represents a positive advancement to the usual approach of describing all observed movements by a single power-law (or other) distribution. However, the state-space approach does not require pre-processing of the data, since the partitioning into different states is part of the overall fitting process.

For example, the use of state-space models to analyse location data from satellite transmitters fitted to grey seals revealed that the seals focussed foraging efforts on a smaller fraction of the continental shelf area than was previously thought [53]. Another recent use [54] revealed migration pathways and multispecies hotspots of marine predators. And biological questions such as how well do animals navigate can be addressed in a quantitative fashion [55]. A Lévy movement model could be tested in the state-space framework as a searching model, and it could be compared with other candidate searching models using a model selection approach. Other recommended modelling approaches include hidden Markov models [56] (a particular class of state-space model) and mechanistic home range models [57], [58], which are biologically intuitive because they emphasize the underlying mechanisms responsible for the observed movement patterns.

Given our findings, we caution against the idea [27] that “Lévy-like walks may be useful for developing more realistic models of how animals redistribute themselves in response to shifting resources as a consequence of climate change, fisheries extractions and other habitat modifications.”. We therefore also discourage the logical extension of such work, which would be to use such models to provide advice to managers of marine ecosystems.

## Methods

Here we briefly discuss Bayesian weights, give the derivations for equations (7) and (8), and document the methods (and associated problems) used to calculate Akaike weights in [28].

### Bayesian Weight Calculations

For method **e** in Table 1, Bayesian, rather than Akaike, weights were calculated in [27]. Bayesian weights are calculated similarly to Akaike weights (e.g. page 290 of [37]), but use the Bayesian Information Criterion (BIC) in place of AIC. The BIC is calculated from the log-likelihood of a model as
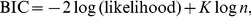
(10)where *n* is the sample size and *K* is the number of parameters being estimated [37]. Whereas AIC is calculated as



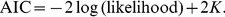
(11)Using the log-likelihood values given in Table 1 for the PL and Exp models, we calculate respective BIC values of 236,272 and 232,615, giving Bayesian weights of 0 and 1, the same as the Akaike weights in Table 1. Thus, Bayesian and Akaike weights give the same results, and so we used Akaike weights (as were mostly used in [27]).

Also, for the small sample 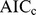 used in [27], the 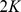 term in (11) is multiplied by 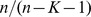. This is essentially 1 for the bigeye tuna data (given 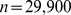 and 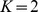 for the two models), and AIC and 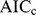 give identical Akaike weights.

### Derivation of Equation (7) for the Exponential Model

As outlined in the main text, movement steps, *x*, were put in descending order such that their respective ranks were given by 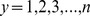; 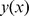 thus represents the number of steps 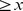. The exponential model was tested in [27] by fitting a straight line to 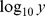 against *x*. Thus,
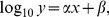
(12)

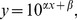
(13)where 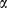 and 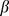 are the fitted coefficients. Since *y* represents the number of steps 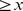, we have


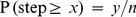
(14)


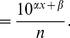
(15)

To derive (7), first note that 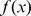 is a probability density function for step sizes. Thus it equals, by definition [38], the gradient of 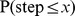, the cumulative distribution function for a step size, which equals the gradient of 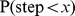 for continuous distributions. Thus, we have
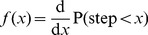
(16)

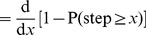
(17)

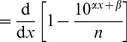
(18)

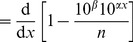
(19)

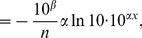
(20)where the last step comes from using the relationship



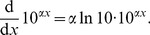
(21)Now define 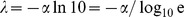, to give
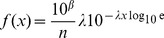
(22)

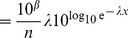
(23)

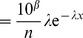
(24)

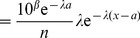
(25)

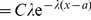
(26)where 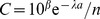, giving the required equation (7).

### Derivation of Equation (8) for the Quadratic Model

We now derive the probability function 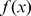 given in (8) that relates to the quadratic model. On page 4 of their Supplementary Information, [27] introduced “a quadratic model 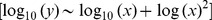 describing intermediate behaviour”. Presumably the final 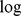 should also be 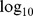 and the term should be 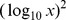.

So a quadratic model was fitted to the data plotted on the rank/frequency plots with 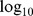 axes. We explicitly write the model fit as
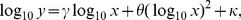
(27)where 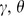 and 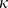 are the fitted regression coefficients. The coefficients are calculated by doing a multiple linear regression [21]. Equation (27) can also be written as



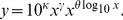
(28)Denoting the probability density function to be 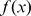, as for (17) we have
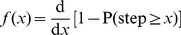
(29)

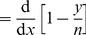
(30)

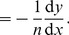
(31)


Using the fact that
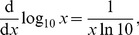
(32)it is easier to differentiate (27) with respect to *x*, rather than to differentiate (28). This gives


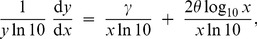
(33)


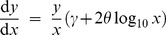
(34)


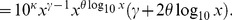
(35)

Substituting into (31) results in
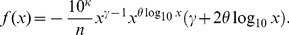
(36)This is the formulation (8) given in the main text.

To see if this is indeed somehow intermediate between power-law and exponential distributions, we now cast it in terms of a power-law term multiplied by a (complicated) exponential term and then multiplied by a 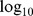 term. Substitute.
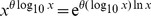
(37)to give
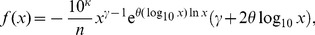
(38)which does not appear to be an intermediate distribution (or even a valid distribution – Issue three).

The normalisation condition can be most simply checked by using the fact that 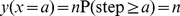. Putting 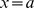 into (28) gives.
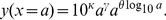
(39)This clearly is not simply *n* (which does not even appear in the equation), showing that 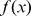 is not properly normalised. Again, this is due to the fact that 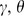 and *k* are determined from a linear regression (multiple linear regression in this case), with no consideration of *a* or *n*.

### Outline of the Akaike Weight Calculations in Ref. [28]

Examination of the detailed Supporting Online Material of [28], email clarifications with the lead author, and examination of the R code used for the analyses (M. de Jager, pers. comm.), determined that the methods used to estimate parameters and calculate likelihoods to compare the fits of models were as follows:

1. Load in the data of step lengths and sort into ascending order.
2. First consider the bounded power-law (PLB) model, as given in (3).
  - Fix the lower bound 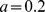, just below the minimum value of the data of 0.21095 (for the data in Fig. 1B of [28]).
  - Create a vector of values of the exponent 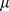 to test, namely (1.1, 1.2, 1.3, …, 5.9, 6.0).
  - Set a value of the upper bound *b*. Steps (c)-(g) will then be repeated for different values of *b*.
  - For each value of 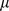 in the above vector, calculate 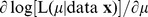, the partial derivative with respect to 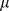 of the log-likelihood function 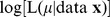, given below in (42).
  - Find the value of 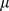 that minimises the absolute value (has the value closest to 0) of 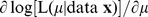. This is the estimated value of 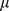 for the given value of *b*.
  - Calculate the fitted inverse cumulative frequency distribution (evaluated for each step in the data set) using the values of 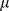 and *b*.
  - Repeat (c)-(f) for incrementally increasing values of *b*.
  - Each value of *b* thus has a corresponding 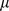 with an absolute value of 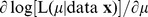 as calculated in (e). Select the *b* that corresponds to the lowest overall absolute value of 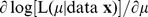. This *b*, and its corresponding 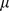, are then considered to be the best fitting values for the PLB model.
3. Calculate the maximum likelihood estimate for 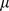 for the unbounded power-law distribution (PL model, equation (1)) from the analytical solution (e.g. [14]). Calculate the corresponding fitted inverse cumulative frequency distribution.
4. Calculate the maximum likelihood estimate for 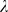 for the exponential distribution (Exp model, equation (2)). Calculate the corresponding fitted inverse cumulative frequency distribution.
5. Calculate an AIC value for each model. The AIC for the PLB model, for example, was calculated in R using the command
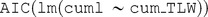

  where 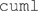 is the observed distribution and 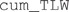 is the fitted distribution in 2(f) calculated for the *b* corresponding to the best fit in 2(h). The observed distribution 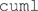 just takes the values 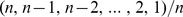 for sample size *n*.
6. Calculate Akaike weights to compare models. This was done using the following R code, where 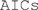 is a vector containing the AIC values for the three models and 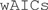 gives the resulting Akaike weights:
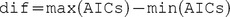


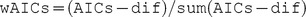

7. Comparisons were also made using G-statistics and the sum of squared differences between the fitted distributions and the observed distribution.

### Problems with the Above Akaike Weight Calculations

We now highlight some problems with the above methods, referencing by step number.

2(b). Only testing discrete values of 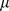 will limit the accuracy of the method, and does not allow for calculation of confidence intervals to yield the associated uncertainty of any estimate.

2(e). Rather than find the closest value of 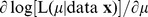 to 0, this gradient term should be set to 0 and solved numerically, to give an exact maximum likelihood estimate for 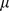. This avoids the need to specify discrete values in step 2(b).

2(h). Selecting the *b* corresponding to the lowest absolute value of 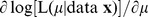 is just selecting the *b* for which the derivative of the log-likelihood function happens to get closest to 0 (because it is only calculated at discrete values of 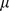). This is not the same as determining which value of *b* gives the maximum overall likelihood. In fact, it can be shown analytically (below) that setting *b* to be the maximum value in the data set will maximise the likelihood. So there is actually no need to test multiple values of *b* to maximise the likelihood.

4 The equation in the code incorrectly assumed the exponential distribution to reach 0, but if the power-law distributions are assumed to start at *a* then the exponential distribution should also start at *a*, and the equations given in [14] should be used. See [8] for other published examples of this exact issue.
5 The AIC calculation is based on linear fits of models – this is Issue one discussed above with respect to [27]. The details are slightly different, but the main message is the same.
6 Even if the above issues did not hold, the code for the Akaike weights is incorrect (e.g. see equation (8) in [14]). Correct code to calculate the vector of Akaike weights 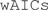 from the vector of AIC values 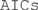 is:
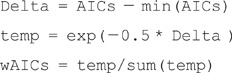

7 The use of the additional methods involving G-statistics and sums of squares is not justified. In Issue one, equation (6) shows that minimising the sum of squares should give the same result as maximising the erroneous likelihood, so there is no need for such an extra method.

Some of the above problems (and others) were independently raised in [31], and addressed in [30].

### Derivation of Maximum Likelihood Estimate of *b* for the PLB Model

Regarding the above Step 2(h) of the methods of [28] when fitting the PLB model (3), we now show that the likelihood function is maximised when setting the bound *b* equal to the maximum value in the data set.

Given a data set 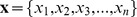, and requiring *b* to be estimated, for 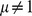 the log-likelihood function is (equation (A.23) of [8]):
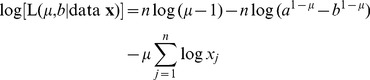
(40)

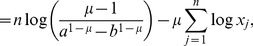
(41)where 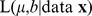 is the likelihood of the unknown parameters 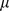 and *b* given the known data **x** (assuming *a* is fixed). Equations (40) and (41) are equivalent, though the form (40) cannot be evaluated for 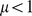. The partial derivative with respect to *b* is



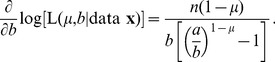
(42)For 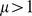, the numerator is negative and the denominator is positive (since 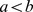), so (42) is 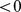. For 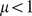, the numerator is positive and the denominator is negative, so again (42) is 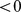.

For 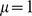, the log-likelihood function is (equation (A.25) of [8]):
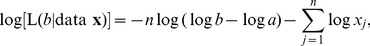
(43)for which



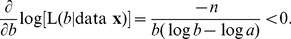
(44)Therefore the partial derivative is always negative, and so the log-likelihood is maximised for the smallest possible value of *b*. By definition this is the maximum value of the data set being fitted to. Similar calculations show that if *a* is to be estimated, then the maximum likelihood estimate of *a* is the minimum value of the data set.

On page 39 of the Supplementary Information of [33] it was stated that the likelihood function of the PLB model cannot be calculated for 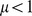. This is not so – see (41) and [15]. Table S2 of [33] gave an equation related to the maximum likelihood estimate for 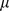 for the PLB model, taken from Table 1 of [15]. Ref. [15] stated that the equation is valid for 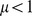, so it is unclear why [33] could not compute it for 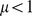. Perhaps it was because the equation in Table S2 of [33] is not the same as that in [15] (it has been incorrectly re-arranged, and *y* is not defined).

### Goodness-of-fit Tests for Mussels Data Set

To test whether the corrected mussels data set from [30] is indeed consistent with coming from the PLB model, we conducted goodness-of-fit tests using the G-test (likelihood-ratio test) with Williams’s correction [42], as in [14]. Parameter *a* was fixed at 0.2 (as in [30]), *b* was estimated as the maximum step length in the data set (119.1893 mm), the sample size 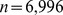 and the MLE for 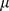 for the PLB model is 1.87. The two binning procedures described in Appendix A of [8] were used, here named Protocol 1∶ bin widths of 1 and then doubling the bin width once <5 data points were in a bin, and Protocol 2∶ doubling the bin widths straight away (i.e. bins of 1, 2, 4, 8, …; and doubling again if there were <5 data points in a bin). Protocol 1 resulted in 23 degrees of freedom (dof), goodness-of-fit value 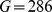, and 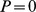, thus the data are not consistent with the PLB model (if 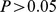 then we would have concluded that the data are consistent with the model at the 0.05 level [42]; this would have required that 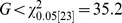, where 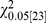 is the value to the right of which is found 0.05 of the area under a 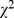 distribution with 23 dof [42]). Protocol 2 resulted in the same conclusion (with 3 dof, 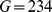 and 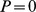). Given that a bin width of 1 resulted in most of the data points ending up in the first bin, we repeated the analyses with initial bin widths of 0.1 and 0.01, to see if our results were dependent on the bin widths (a bin width of 0.01 results in just 3.3% (234/6996) of the data in the first bin). The results were (i) with the first bin width of 0.1 (Protocol 1∶ 96 dof, 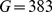 and 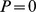; Protocol 2∶ 7 dof, 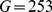 and 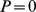), and (ii) with the first bin width of 0.01 (Protocol 1∶ 205 dof, 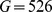 and 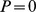; Protocol 2∶ 10 dof, 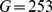 and 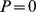). Thus the conclusion of 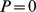 is robust to the binning procedure, and the data are definitively not consistent with the PLB model.

Note that although AME was thanked for “comments and suggestions” in [32] and had corresponded with two of the authors, he had not seen an earlier version of [32] nor was aware of its content or existence until it was published. Also note that our re-analysis is performed on the data set of 6,996 values that appears in the Erratum [30], corrected from the original in [30], yet this is different to the corrected data set provided to the authors of the Technical Comment (see Note 10 in [31]).

## Supporting Information

Code S1 **R code for standard calculations of likelihood and Akaike weights for bigeye tuna data.**
(R) Click here for additional data file.

Code S2 **R code for calculations regarding Issues one to three, for bigeye tuna data.**
(R) Click here for additional data file.

Code S3 **R code for goodness-of-fit calculations for both data sets (called from Code S1 and Code S4).**
(R) Click here for additional data file.

Code S4 **R code for standard calculations of likelihood and Akaike weights for mussels data.**
(R) Click here for additional data file.

Code S5 **R code to calculate summary statistics for range calculations related to** [**33**] **.**
(R) Click here for additional data file.

Code S6 **Pseudo data file based on the original bigeye data, obtained by sampling (with replacement) the original step sizes to obtain a pseudo data set with similar properties.**
(TXT)

Code S7
**Pseudo data file based on the original mussels data, obtained by sampling (with replacement) the original step sizes (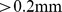) to obtain a pseudo data set with similar properties.**
(TXT)
